# Emerging Cell-Based Therapies for Systemic Sclerosis: From Stem Cells to CAR-T Cells

**DOI:** 10.3390/cimb48010076

**Published:** 2026-01-12

**Authors:** Vitaly Chasov, Sabir Mukhametshin, Elvina Gilyazova, Damir Davletshin, Mariya Tikhomirova, Iuliia Topchu, Aygul Valiullina, Marcella Prete, Emil Bulatov

**Affiliations:** 1 Institute of Fundamental Medicine and Biology, Kazan Federal University, Kazan 420008, Russia; 2 Internal Medicine Unit, Department of Interdisciplinary Medicine, Aldo Moro University of Bari Medical School, 70124 Bari, Italy; 3 Shemyakin-Ovchinnikov Institute of Bioorganic Chemistry, Russian Academy of Sciences, Moscow 117997, Russia

**Keywords:** systemic sclerosis, autoimmune diseases, cell-based therapies, hematopoietic stem cell transplantation, mesenchymal stem cells, CAR-T cell therapy

## Abstract

Systemic sclerosis (SSc) is a disease in which malfunctioning immune cells lead to the formation of autoantibodies that damage blood vessels and body tissues. Fibrosis then develops in the affected organs. Its complex pathogenesis involves multiple immune and stromal cell types, soluble mediators, and dysregulated tissue repair, resulting in heterogeneous clinical manifestations and poor prognosis. Current disease-modifying therapies provide only modest benefits, often slowing but rarely reversing disease progression, and are associated with considerable adverse effects. These limitations have spurred the development of cell-based therapeutic strategies aimed at restoring immune tolerance and promoting tissue repair. In this review, we summarize recent advances in hematopoietic stem cell transplantation, mesenchymal stem cell therapy, and adoptive regulatory T cell transfer and highlight the emerging role of chimeric antigen receptor (CAR)-T cell therapy as a transformative approach for SSc. Collectively, these evolving strategies hold the potential to improve survival, achieve durable remissions, and significantly enhance quality of life for patients with SSc.

## 1. Introduction

The hallmarks of SSc, a chronic autoimmune disease, are connective tissue deposition, endothelial cell injury, progressive fibrosis, vasculopathy, immune dysfunction, inflammation and autoantibody production [1]. The etiopathogenesis of the disease is complex, but significant progress has been made in understanding the mechanisms involved: genetic predisposition and environmental factors lead to cytokine release, immune cell activation, and dysregulation of connective tissue repair [2,3,4]. A variety of cellular components are involved, primarily endothelial cells, mesenchymal cells, and T and B lymphocytes, whose activation leads to the release of proinflammatory cytokines (IL-6, IFN-alfa, IL-10, TGF-beta, PDGF) [5]. It affects multiple organs and systems, such as the skin, lungs, kidneys, heart, gastrointestinal tract, and musculoskeletal system [6]. Raynaud’s phenomenon is one of the main symptoms of SSc, which is characterized by impaired blood circulation in the fingers and toes, caused by the spasm of small blood vessels in response to emotional stress, cold temperatures, or other influences. It is often accompanied by the development of digital ulcers. [7]. In addition to extensive fibrosis of the internal organs and skin, pulmonary arterial hypertension—another manifestation of vasculopathy—can lead to disability and death in patients with SSc [8,9]. In SSc, myofibroblasts produce excessive amounts of collagen and other extracellular matrix proteins in response to damage to the connective tissue, thereby causing changes called fibrosis [10].

Currently, SSc is considered incurable. Treatment for SSc remains a challenge due to the unclear pathogenesis [10]. Patients with SS are usually prescribed immunosuppressive medications as a standard treatment method, which carry the risk of developing side effects with long-term use, such as increased susceptibility to infections and even the development of cancer [11,12,13]. Unfortunately, standard therapy does not have a long-term effect and usually only slow the disease progression and rarely reverse disease manifestations, thus increasing the mortality rates [8,9]. Therefore, there is a need for the development of new therapeutic approaches that can restore self-tolerance before the onset of extensive and irreversible damage as a result of SSc.

**Figure 1:**
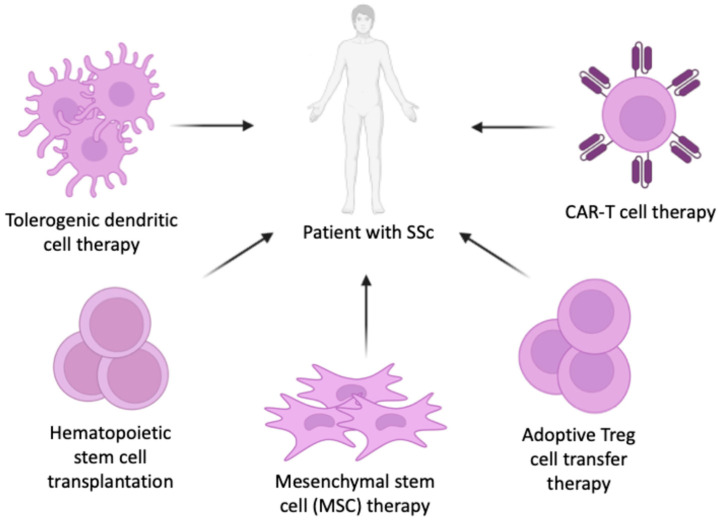
Overview of cell-based therapeutic strategies under investigation for systemic sclerosis (SSc). Approaches include hematopoietic stem cell transplantation (HSCT), mesenchymal stem cell (MSC) therapy, tolerogenic dendritic cell (TolDC) therapy, adoptive regulatory T (Treg) cell therapy, and chimeric antigen receptor T (CAR-T) cell therapy. These interventions aim to restore immune tolerance, reduce fibrosis, and improve clinical outcomes in patients with SSc.

Researchers have high hopes for cell-based therapy methods, as they have demonstrated significant promise in treating SSc [14]. Here, we review the available data on cell-based therapies for SSc, such as hematopoietic stem cell transplantation (HSCT), mesenchymal stem cell (MSC) therapy, adoptive regulatory T cell (Treg) therapy, and CAR-T cell therapy (Figure 1). Tolerogenic dendritic cell (TolDC) therapy is presented here simply as a promising idea for future research. Understanding the advantages and disadvantages of each allows us to select the most promising ones for further study and eventual translation into clinical practice. In the near future, they may be able to help patients with SSc achieve stable remissions, prolong survival, and significantly improve their quality of life in the long term.

## 2. Methods: Identification of Clinical Trial Data and Evidence Synthesis for Table 1 and Table S1

To compile Table 1 and Supplementary Table S1, we performed targeted searches of clinical trial registries and the peer-reviewed literature focusing on systemic sclerosis (SSc) cell-based interventions. Clinical trial records were retrieved from ClinicalTrials.gov (NCT identifiers) using the terms “systemic sclerosis” OR “scleroderma” combined with therapy-specific keywords (“stem cell transplant”, “mesenchymal stromal/stem cell”, “regulatory T cell”, “tolerogenic dendritic cell”, “CAR T”, and “chimeric antigen receptor”). The last registry search was performed on 10 December 2025; records from database inception through this date were considered.

Eligible registry records were interventional studies enrolling patients with SSc (including juvenile SSc) and evaluating a cell-based therapy (HSCT, MSCs, adoptive Treg transfer, TolDC therapy, or CAR-based cellular products). Mixed-disease studies were included only when SSc constituted an explicitly listed cohort/stratum; such entries are labeled accordingly. To capture published outcomes and safety data (including case reports), we searched PubMed/MEDLINE and screened reference lists of relevant reviews and primary studies (database inception through 10 December 2025). For trials with multiple reports, we cited the most recent peer-reviewed publication. Trial characteristics and, where available, key efficacy endpoints and adverse events were extracted for Table S1; Table 1 provides a condensed overview. Where outcomes remain pending, entries are marked as “ongoing or N/A”.

**Table 1:** Condensed overview of registered clinical trials of cell-based therapies for systemic sclerosis (SSc), identified from ClinicalTrials.gov (NCT identifiers, last accessed 10 December 2025). Sample size and phase are reported as stated in the registry. Detailed outcomes, adverse events, and supporting citations are provided in Supplementary Table S1.

Therapy/Target	Patients (n)	Trial ID	Phase of Trial
Autologous CD34^+^ HSCT (myeloablative, CD34-selected) vs. 12 × monthly CYC	75	NCT00114530	III
Autologous non-myeloablative HSCT vs. cyclophosphamide (CYC)	19	NCT00278525	II
Autologous HSCT (various conditioning)	82	NCT02516124	N/A
Allogeneic bone marrow derived MSC vs. placebo	20	NCT03211793	II
Toxicity and efficacy of allogenic MSC for severe SSc vs. CYC	20	NCT02213705	II
Safety and efficacy of allogeneic MSCs	14	NCT00962923	II
Efficacy and safety of adipose-derived-MSC vs. placebo	32	NCT04356755	II
Compare single vs. repeated umbilical cord-derived MSC infusions vs. placebo	18	NCT04356287	II
Polyclonal autologous CD4^+^CD25^+^ Tregs to study immune suppression/tolerance	25 (15 Treg, 10 control)	NCT05214014	II
KYV 101, an autologous CD19 CAR-T cell therapy	21	NCT06400303	II
Allogeneic CD19/BCMA-targeted CAR-T cell therapy	12	NCT06941129	I
CD19/BCMA-targeted CAR-T cell therapy	9	NCT05085444	I
Allogeneic CD19-targeted CAR-T (CT1190B)	27 (SSc and SLE)	NCT06822881	I
Anti-CD19 CAR-T (juvenile SSc)	12	NCT06792344	I
CD19/BCMA-targeted CAR-T cell therapy	50 (multiple diseases)	NCT06794008	II
Allogeneic CD19-targeted CAR-T cell therapy	3 (2 SSc, 1 myositis)	NCT05859997	N/A
A single dose of autologous CD19-targeted CAR-T cell (CABA-201) in combination with CYC and fludarabine	12	NCT06328777	II

Notes: Patient numbers correspond to planned enrollment unless otherwise stated. Abbreviations: HSCT, hematopoietic stem cell transplantation; CYC, cyclophosphamide; MSC, mesenchymal stromal/stem cells; Treg, regulatory T cells; CAR-T, chimeric antigen receptor T cells; SLE, systemic lupus erythematosus; N/A, not available/not reported.

## 3. Hematopoietic Stem Cell Transplantation

HSCT is a multi-step procedure that replaces a patient’s hematopoietic system with hematopoietic stem cells (HSCs). It is traditionally used to treat hematological malignancies but has also been shown to be effective in treating autoimmune diseases [15]. HSCs can be obtained from two different sources: a healthy donor (allogeneic HSCT) or the same patient (autologous HSCT). For a more successful outcome, CD34 selection is required to enrich HSCs and remove autoreactive lymphocytes, which is achieved by a conditioning regimen, for example, with anti-thymocyte globulin (ATG) [16,17].

In recent decades, the potential of HSCT for treating patients with SSc has been intensively studied (Table 1) [17,18,19,20,21]. According to a preliminary phase I/II clinical trial, there was a trend toward improvement in skin condition (69%) after the treatment of 41 patients with SSc with autologous HSCT [22]. However, lung function did not change significantly, and there was increased mortality (17%), which was considered to be related to the procedure (direct organ toxicity, hemorrhage, and infection/neutropenic fever) [22]. Then, a phase I/II clinical trial was conducted on 12 patients with SSc to evaluate the feasibility and tolerability of autologous HSCT. The results indicate that eight out of eleven patients achieved a major or partial clinical response, and there was one procedure-related death [18]. A number of randomized controlled trials have also been conducted comparing autologous HSCT with standard immunosuppressive therapy in SSc (Table 1). In Phase II of the trial (NCT00278525), nineteen people diagnosed with SSc were randomly assigned to two groups [19]. The first group received non-myeloablative HSCT without prior modification, while nine patients in the second group received monthly cyclophosphamide injections [19]. After one year, all patients in the HSCT group experienced cutaneous and pulmonary improvements, whereas there was no significant long-term benefit after standard cyclophosphamide immunosuppression [19]. In the phase III ASTIS trial, a total of 156 patients with early diffuse cutaneous SSc were randomized to receive HSCT or cyclophosphamide as standard immunosuppression (ISRCTN54371254) [20]. This study showed that HSCT was associated with increased treatment-related mortality in the first year after treatment (16% vs. 10.4% in the control group); however, at a median follow-up period of 5.8 years, HSCT demonstrated a significant improvement in long-term overall survival compared to intravenous pulse cyclophosphamide [20]. In addition, 75 patients with severe SSc were randomized to receive myeloablative CD34+ selected autologous HSCT (36 participants) or monthly infusions of cyclophosphamide (39 participants) (NCT00114530) [17]. The study results showed that the event-free survival rate at 54 months was 79% in the HSCT group, compared to 50% in the cyclophosphamide group. At 72 months, the event-free survival rate was 74% vs. 47%. The overall survival rate was 86% vs. 51% [17]. Thus, HSCT demonstrated long-term benefits for patients with SSc, including improved event-free and overall survival [17]. After that, a global transcript study was conducted using Illumina HT-12 arrays to determine the molecular changes in whole blood transcripts and serum protein levels resulting from HSCT, as compared to monthly intravenous cyclophosphamide [23]. This study measured the levels of 102 proteins in serum samples collected from 62 participants [23]. A paired comparison of samples from 26 months and the baseline demonstrated that the interferon and neutrophil transcript modules decreased significantly, while the cytotoxic/natural killer (NK) cell module increased significantly in HSCT group, while there was no significant change in the cyclophosphamide group, suggesting that the therapeutic effect of HSCT in the treatment of SSc is associated with profound changes at the molecular level [23]. In addition, a prospective, non-interventional study was conducted to evaluate the efficacy and safety of HSCT in treating severe SSc in 80 patients (NCT02516124) [21]. The study’s findings revealed that after two years, 81.8% of patients remained progression-free, 90% survived overall, 88.7% showed a positive response, and 11.9% experienced progression. The 100-day non-relapse mortality rate was 6.25% [21]. This study confirms the efficacy of autologous HSCT for severe SSc using nonmyeloablative conditioning in real-life practice. In a recent study, the safety and long-term effectiveness of autologous HSCT was evaluated in 17 patients with severe SSc [24]. Improvements in skin sclerosis, pulmonary function, and gastrointestinal symptoms were observed [24]. The overall survival rate was 94.2% over the 9.1-year median follow-up period [24]. Based on the aforementioned clinical trials, the European Society for Blood and Marrow Transplantation currently recommends autologous HSCT as the standard treatment for refractory SSc [25]. However, as the mortality rate associated with HSCT treatment for SSc is quite high, this therapy is only recommended for patients with an unfavorable prognosis [26].

## 4. Mesenchymal Stem Cell Therapy

Another cellular therapy approach is MSC therapy. MSCs are a heterogeneous population of adult multipotent stem cells with a fibroblast-like morphology and the ability to differentiate into multiple tissue lineages [27]. In addition, these cells have a high regenerative capacity, which allows them to be isolated, cultured and expanded ex vivo, and their immunosuppressive, angiogenic and anti-fibrotic properties are finding application in the treatment of many autoimmune diseases, including SSc [27]. The immunomodulatory properties of MSCs are specifically characterized by their ability to promote an anti-inflammatory phenotype, in particular by decreasing the populations of DCs, macrophages, B and T cells, NK cells, and by promoting the production of Tregs and the release of IL-10, hepatocyte growth factor, transforming growth factor (TGF)-β, prostaglandin E2 (PGE2), the soluble form of the protein HLA-G5, nitric oxide (NO) and indolamine-2,3-dioxygenase (IDO), all of which play a role in the suppression and regulation of inflammatory responses [28,29]. A special trial using a single dose injection of bone marrow-derived MSCs (BM-MSCs) in 19 patients with severe SSc confirmed the immunomodulatory properties of MSC therapy (NCT02213705) [30]. The study included 14 patients who responded to the treatment and 5 who did not respond. The researchers characterized the circulating immune cells of the patients to further understand the effects of MSCs on the immune system [30]. An increase in the number of circulating memory B cells, the main IL-10-producing regulatory B cell subset (Bregs) was observed, as well as an upregulation of IL-10 expression in purified B cells ex vivo, specifically in responder patients, shortly after the BM-MSC infusion [30]. The ability of BM-MSCs to directly upregulate IL-10 production in activated B cells was then confirmed in vitro [30].

In recent years, there has also been great interest in exosomes, the small extracellular vesicles secreted by MSCs for intercellular communication, which have low immunogenicity, contain microRNA (miRNA), long non-coding RNA (lncRNA), small interfering RNA (siRNA), DNA and protein, and therefore have great potential for therapeutic applications [31,32]. According to the recommendations of the International Society for Cellular Therapy (ISCT), the minimum criteria for the use of MSCs in clinical practice are: the ability to multilineage differentiation, the ability to adhere to plastic surfaces, positive expression of surface markers CD105, CD73 and CD90 and negative expression of surface markers CD45, CD34, CD14, CD19 and HLA-DR [33]. Using a bleomycin-induced SSc model, the importance of preclinical conditioning of MSCs was also noted [34]. Specifically, IFN-γ- and TNF-α pretreatment of MSCs has been shown to significantly reduce skin fibrosis by reducing the accumulation of macrophages, known to be the predominant profibrotic immune cell population in the pathogenesis of SSc [34].

MSC therapy has already been successfully used to treat other autoimmune diseases such as systemic lupus erythematosus (SLE), type 1 diabetes and rheumatoid arthritis (RA) [35,36,37,38]. There are data on the use of both autologous and allogeneic MSCs in SSc patients. For example, a 34-year-old patient with critical limb ischemia and acute gangrene due to SSc, refractory to all conventional first-line treatments, was treated with 3 intravenous pulses of expanded autologous MSCs [39]. The results of the study showed that the first infusion of MSCs reduced skin necrosis, and after the third infusion angiography showed new vessel formation [39]. Eight patients with refractory ulcers caused by SSc were then enrolled to evaluate the efficacy and safety of autologous transplantation of bone marrow-derived MSCs isolated and injected into skeletal muscle of the ischemic limb [40]. A reduction in the size and number of ulcers, an increase in blood flow and new capillaries in the nail bed were observed in all patients after treatment [40]. Importantly, there were no major adverse effects associated with this treatment [40].

The study of transplantation of bone marrow-derived MSCs from an allogeneic haploidentical related donor into a 41-year-old female patient with diffuse cutaneous SSc showed a significant reduction in the patient’s painful ulcerations 3 months after transplantation, while vascular ultrasound examination 6 months after treatment showed a significant improvement in hand and finger circulation and angiography confirmed revascularization [41]. Based on this study, a conclusion was drawn regarding the feasibility, safety and efficacy of this procedure [41]. The same group of researchers then carried out a study of five patients with severe SSc who were followed for between 6 and 44 months, although the location of the lesions and the degree of organ damage varied between patients [42]. The study showed that the most pronounced therapeutic effect after transplantation of MSCs from allogeneic related donors was observed in the healing of skin ulcers, but the heterogeneity of disease symptoms among these patients made it impossible to draw a clear general conclusion about the effectiveness of the procedure [42]. However, MSC transplantation did not lead to immediate toxicity or the development of serious infections [42].

A phase I-II trial aimed to evaluate the safety and efficacy of a combination of plasmapheresis and allogeneic umbilical cord (UC) MSC transplantation (NCT00962923) (Table 1) [43]. The trial began in 2009 and studied 14 patients with SSc, who received three courses of plasmapheresis, followed by pulse cyclophosphamide on days 1, 2, and 5 [43]. UC-MSCs were infused on day 8. At 12 months, patients had improved modified Rodnan skin score (MRSS) and significantly reduced levels of Scl70 antibody, serum transforming growth factor-β (TGF-β) and vascular endothelial growth factor (EGF) [43]. In addition, patients with interstitial lung disease had improved lung function and computed tomography scans [43]. Among other clinical trials using MSCs to treat SSc, the following should be noted (Table 1). A phase I–II study evaluating the toxicity and efficacy of allogeneic MSC therapy for the treatment of severe, rapidly progressive or cyclophosphamide-refractory SSc has been completed with an unknown outcome (NCT02213705). The Phase I–II study (NCT03211793) is now recruiting patients with SSc and digital ischemia with refractory ischemic digital ulcers that are refractory to conventional treatment. Local toxicity will be assessed, including signs of local inflammation (swelling, fever, dysfunction), worsening of ulcers or appearance of new ulcers or hematomas after allogeneic BM-MSC administration, and other adverse events. Patients are also being recruited for the trial to test the safety and efficacy of umbilical cord MSCs (UC-MSCs) for the treatment of SSc (NCT04356287). One study will compare the efficacy and safety of digital injection of cultured adipose-derived mesenchymal stem cells (AD-MSC) with placebo (NCT04356755). There has also been a trial using autologous adipose-derived regenerative cells (ADRCs) to improve hand function in patients with SSc (NCT02396238) [44]. The results show that hand function has improved and the ADRC treatment was well tolerated [44].

The long-term survival rate among SSc patients following MSC therapy was also assessed in 333 individuals, comprising 113 who received MSC treatment and 220 who were in the control group [45]. The investigation revealed that the 10-year cumulative survival rate was 89.4% among those who received MSC therapy compared to 73.4% for those in the control group [45]. Consequently, another significant benefit of MSC treatment is the substantial improvement in the survival chances of SSc patients.

Taking all of the above into account, it can be concluded that MSC therapy shows great promise as a treatment for SSc. However, there are several risks associated with MSC therapy, including infections, graft-versus-host disease (GVHD) in allogeneic transplants, and long recovery periods with possible fatigue or organ damage [46]. Additionally, treatment using MSCs is more expensive, so it is advisable to use it to treat patients with SSc who do not respond to immunosuppressant drugs and experience side effects from long-term use of such therapy.

## 5. Tolerogenic Dendritic Cell Therapy

Dendritic cells (DCs) are a special type of antigen-presenting cell that plays an important role in the immune system, and immature DCs can be activated in two ways: towards mounting an immune response, or towards tolerance (TolDCs) [47]. TolDCs promote immune tolerance by expressing inhibitory molecules and secreting anti-inflammatory cytokines (e.g., IL-10) during antigen presentation, so that the interaction between T cells and TolDCs can lead to either antigen-specific T cell deletion or their differentiation into Tregs, depending on the nature of the intercellular signals [47]. As a result, TolDCs can be used as therapeutic agents for the treatment of autoimmune diseases; when loaded with disease-specific target antigens, TolDCs are able to correct a dysfunctional immune system by targeting T cells [48]. After infusion, TolDCs can be detected in several organs, including the liver, lungs, spleen, kidneys, thymus, bone marrow, lymph nodes, and central nervous system; however, it should be noted that the anti-inflammatory properties and ability to migrate to target organs are affected by different methods of obtaining and administering TolDCs [49].

TolDCs therapy has been successfully used to treat multiple sclerosis (MS) in EAE-induced mice [50]. Infusion of bone marrow-derived TolDC cultured in the presence of vitamin D3 and pulsed with myelin oligodendrocyte glycoprotein peptide 40–55 (MOG(40–55)) has been reported to increase the population of Tregs and decrease the clones of autoreactive T cells, leading to neurological improvement [50]. Additionally, TolDC therapy has been studied in clinical trials with patients suffering from MS, RA, and type 1 diabetes [51,52,53]. These studies have shown that TolDC therapy is safe and well-tolerated [51,52,53]. However, optimizing certain parameters is necessary to increase the efficacy of TolDC [51]. Given that these preliminary studies in the above diseases have shown that TolDC administration is generally safe and tends to restore immune tolerance, similar studies in SSc are expected in the near future. As of 2025, no data had been published on preclinical studies or clinical trials of TolDC therapy for patients with SSc. This type of therapy is presented here as a translational concept rather than a clinical option.

## 6. Adoptive Treg Cell Therapy

Tregs are a type of CD4 T helper cell that can control immune responses. They are characterized by their expression of Foxp3, a transcription factor, and their CD25 receptor for IL-2 [54].

In adoptive Treg therapy, Tregs are isolated from the patient, activated and expanded ex vivo, and then infused back into the patient [55]. The function of Tregs to maintain immune tolerance by suppressing aberrant immune stimulation has been shown to be dysfunctional in several autoimmune syndromes, including SSc [56]. According to the available data, a decrease in circulating Tregs in patients correlates with the activity and severity of SSc [57]. Additionally, regression of fibrosis has been reported following infusion of spleen-derived Treg cells in an animal model of bleomycin-induced pulmonary fibrosis [58]. Thus, using Tregs as an adoptive therapy in SSc may become an effective treatment for controlling autoimmune processes.

Previously, autologous adoptive Treg cell therapy was administered to a patient with SLE, leading to an increase in the number of activated Tregs in the skin, resulted in inhibition of IFN-γ-producing Th1 cells, and a shift toward IL-17-producing Th17 cells [59]. Data from phase I clinical trials of Treg therapy for type 1 diabetes and MS have also been obtained [60,61]. These trials showed an absence of side effects after treatment [60,61]. However, they did not allow for a clear conclusion about the efficacy of the procedure [60,61]. This can be determined in subsequent trials with larger patient populations after Treg stability has been improved.

The above studies suggest that Tregs therapeutic strategy may be a viable approach for treating SSc; however, careful evaluation of preclinical studies and further studies in patients with SSc are needed to confirm the safety and efficacy of this therapy. A major concern about Treg therapy is that these cells have the propensity to differentiate into proinflammatory, cytokine-producing T cells in an inflammatory environment. For example, under certain conditions, Treg cells have been shown to transform into Th1, Th2, and Th17 cells [62,63,64]. The effectiveness of treating patients with SSc with autologous Treg cells is currently being studied in a Phase I/II clinical trial (NCT05214014) (Table 1) [65]. The trial involved 25 patients with SSc, 15 of whom received Treg cells in addition to standard treatment, and 10 who received only standard treatment as a control group [65]. Treg cells were obtained from the peripheral blood mononuclear cells of the participants and injected intravenously. After six months, the group that received Treg treatment showed significant improvement in skin elasticity, without any progression of skin thickening [65].

For the use of Treg therapy in clinical practice, it is also possible to expand and activate Tregs using low-dose interleukin-2 (LD-IL2) (Table 1). A previous study with patients suffering from 11 different autoimmune diseases, excluding SSc, confirmed that LD-IL2 significantly increased the number of regulatory T cells and was safe, without activating effector T cells [66]. In this trial, the patients received 1 × 10^6^ international units (IU) of IL2 daily for 5 days, followed by weekly injections for 6 months [66]. A special study was conducted to investigate the effects of LD-IL2 therapy on 66 patients with SSc [67]. As compared to healthy controls, the absolute number of peripheral T cells, CD4+ T cells, CD8+ T cells, NK cells, and Treg cells was significantly lower in SSc patients before LD-IL2 treatment [67]. This resulted in an increased ratio between Th17 cells and Treg cells [67]. After 24 weeks of LD-IL2 injection, the balance between these two types of immune cells was restored, and disease activity was reduced without obvious adverse effects [67]. The next study aimed to assess the safety and biological efficacy of IL-2LD in patients with SSc [68]. LD-IL2, at a dosage of 1 × 10^6^ IU per day, has been shown to selectively activate and expand Tregs [68]. LD-IL2 therapy was well-tolerated, and no serious adverse events were related to the treatment [68].

## 7. CAR-T Cell Therapy

**Figure 2:**
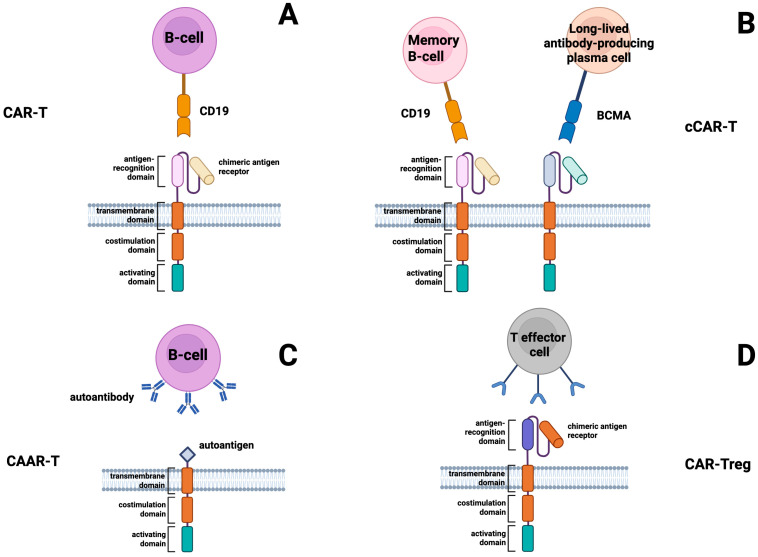
Schematic representation of four CAR-based approaches under investigation for systemic sclerosis (SSc). Panel (**A**)—conventional CAR-T cells targeting CD19 on B cells, leading to cytotoxic elimination of autoreactive populations. Panel (**B**)—compound CAR-T cells (cCAR-T) engineered with dual receptors for CD19 and BCMA, enabling simultaneous targeting of memory B cells and long-lived plasma cells. Panel (**C**)—chimeric autoantibody receptor T cells (CAAR-T), which use disease-relevant autoantigens to selectively eliminate autoreactive B cells producing pathogenic autoantibodies. Panel (**D**)—CAR-T regulatory cells (CAR-Tregs), designed to recognize specific antigens and exert immunosuppressive functions, thereby restoring immune tolerance.

Depletion of autoimmune CD20+ B cells was observed to be an insufficient treatment for SSc, and plasmablasts, which are responsible for producing autoantibodies, cannot be targeted via CD20, but can be targeted by CD19 [69]. Therefore, targeting CD19+ B cells may allow a deep and more tolerable reset of the immune system to treat SSc. From this perspective, the utilization of CAR-T cells appears to be a promising solution (Figure 2). CAR-T cells are engineered T cells that are modified with a specialized gene, known as a chimeric antigen receptor (CAR) [70]. This gene is inserted into the T cells, enabling them to recognize a specific target on the surface of B cells. In this particular case, the target is CD19 (Figure 2A). There is also a type of CAR-T cells, compound CAR-T (cCAR-T) cells, designed for dual targeting of CD19 on B cells and B cell maturation antigen (BCMA) on long-lived plasma cells (Figure 2B) [71]. The depletion of long-lived plasma cells is important in the treatment of autoimmune diseases, including SSc, because these cells are able to survive and produce autoantibodies after the destruction of autoreactive B cells [72]. A recent study using anti-CD19-targeted CAR-T cells to treat five patients with SLE suggests that CAR-T cell therapy may be effective in treating not only cancer, but also autoimmune diseases such as SSc [73,74].

The undesirable consequences of graft-versus-host disease can be prevented by using autologous T cells to obtain CAR-T cells [75]. In autologous CAR-T cell therapy, the original T-cells are taken from the patient’s own body. A patient with severe refractory SSc with fibrosis of the skin, lung and heart was the first patient to receive anti-CD19 CAR-T cells for the treatment of SSc [69]. Three months after treatment began, there was a significant improvement in wrist arthritis, as well as an inclination toward improvement in skin, heart, and joint fibrosis, with less frequent and less severe Raynaud’s phenomenon attacks [69]. These results were the first data indicating the applicability of anti-CD19 CAR-T cell therapy in treating severe SSc that does not respond to other therapies (Table 1). Thus, they paved the way for future studies to confirm the clinical efficacy and long-term effects of anti-CD19 CAR-T cells.

Another case study involved a patient with Scl70+ SSc and rapid progressive nonspecific interstitial pneumonia (NSIP) who received anti-CD19 CAR-T cells [76]. Following treatment, the patient demonstrated regression of skin fibrosis, as assessed by the modified Rodnan skin score (mRSS), as well as dramatic improvement in pulmonary fibrosis [76]. Meanwhile, C-reactive protein (CRP), high-sensitivity troponin T (hsTNT), and anti-topoisomerase I (Scl-70) were normalized or decreased significantly [76]. Remarkably, no severe adverse events were reported [76]. Notably, this study observed a long-term effect for the first time, as CAR-T cells persisted for 11 months and autoantibodies continued to decrease [76].

Six patients with severe diffuse SSc were then recruited to receive anti-CD19 CAR-T cell treatment [77]. Afterward, a detailed analysis of the effects of CAR-T cell therapy on organ manifestations was conducted [77]. The key outcome measures included laboratory parameters, data in the modified Rodnan Skin Score (mRSS), a version of the American College of Rheumatology Composite Response Index in Systemic Sclerosis (ACR-CRISS), lung fibrosis imaging, and patient self-report. Assessments were conducted at baseline and at 3, 6, 9, and 12 months [77]. This research study revealed that, over the course of 487 days, there was no progression of organ manifestations or new events in the lungs, heart, or kidneys [77]. All six patients saw a decrease in skin lesion scores and a significant decrease in median mRSS over 100 days after CAR-T cell treatment. It remained stable or decreased further over one year. The probability of improvement in the ACR-CRISS score increased to a median of 100% over 6 and 12 months compared with baseline [77]. The findings indicate that anti-CD19 CAR-T cell therapy has the potential to reverse organ fibrosis in patients with SSc.

Allogeneic CAR-T cell therapy uses T cells from a healthy donor instead of the patient’s own cells, which holds great promise for increasing the accessibility of CAR-T cell therapy. However, when these cells are administered to a patient, it is crucial to prevent the patient’s body from rejecting them [75]. A recent report, however, showed that allogeneic multi-edited anti-CD19 CAR-T cells (TyU19 cells), generated by CRISPR/Cas9, were used to treat two patients with diffuse cutaneous SSc [78]. A corresponding clinical trial is planned (NCT05859997) (Table 1). The authors report that B cells were completely depleted within two weeks after treatment initiation, while CAR-T cells remained viable for more than three months [78]. Deep remission was observed for six months, evidenced by a significant reduction in inflammation and fibrosis, as well as a significant improvement in clinical response index scores [78]. There were no serious adverse events [78]. This study demonstrated the efficacy and safety of allogeneic CAR-T cell therapy for SSc, which could make CAR-T cell therapy more accessible to patients. However, these findings should be confirmed by other studies and clinical trials.

Then, the same research team developed induced pluripotent stem cell (iPSC)-derived CAR natural killer (CAR-NK) cells (QN-139b) and tested their effectiveness and safety in a patient with severe refractory SSc [79]. QN-139b is a dual-targeting cell therapy for eliminating B cells and plasma cells (CD19/BCMA) in patients with a heterogeneous disease [79]. The researchers reported that QN-139b has a better potential for clonal expansion and an improved safety profile compared to other CAR-T cell therapies, as these cells are genetically modified to reduce the risk of allogeneic rejection and increase their persistence in vivo [79]. The results showed clinical improvements in patients during the 6-month follow-up, including the elimination of autoreactive B cells, suppression of inflammation and fibrosis, enhanced tissue regeneration, improved angiogenesis and restoration of skin and microvascular structure [79].

There are two other novel types of CAR-T cells that could be used to treat SSc (Figure 2). One of these is chimeric autoantibody receptor (CAAR)-T cells, which contain an autoantigen on their receptor and exert a cytotoxic effect due to their affinity for autoantibodies on the surface of B cells (Figure 2C). This allows CAAR-T cells to selectively destroy only autoreactive B cells. As anti-CD19 CAR-T cells deplete both normal and autoreactive B cells, CAAR-T cells that specifically target only the B cells secreting autoantibodies are considered safer and more effective [80]. CAAR-T cells were successfully tested for the first time in a mouse model of pemphigus vulgaris (PV) [81]. This study demonstrated that CAAR-T cells expressing the PV desmoglein 3 (Dsg3) specifically recognize and eliminate anti-Dsg3 target cells [81].

In autoimmune B-cells of SSc, it is important to identify the most appropriate targets for CAAR-T cell therapy [82]. The first candidate is autoreactive B-cells against topoisomerase I, since they, together with their associated immune complexes, can activate myofibroblasts [83,84]. Other autoantibodies that could be useful targets include antibodies against angiotensin receptor type 1 (AT1R), endothelin receptor type A (ETAR), CXCL-4, and platelet-derived growth factor (PGDF) receptor [85,86,87].

Since Tregs are typically suppressed in SSc, an effective approach to restoring immune tolerance is Treg cell therapy [57]. Antigen-specific Treg cells have been found to control autoimmune responses more effectively than polyclonal Treg cells in animal models [88,89]. In addition, using antigen-specific Tregs was found to have advantages over using polyclonal Tregs, as fewer cells are needed and the risk of nonspecific immune suppression is reduced [90]. However, directly isolating antigen-specific Tregs from polyclonal populations is currently challenging due to the limited clonal diversity of the Treg pool and the difficulty of expanding them ex vivo [91]. Therefore, generating antigen-specific CAR-Tregs could be a promising alternative to polyclonal Tregs (Figure 2D) [92]. Without causing systemic immunosuppression, CAR drives Tregs to the location of autoimmune activity, enhancing their suppressive capacity [93]. To obtain the most stable phenotype and expand the cell population, the most optimal way to generate CAR-Tregs appears to be to use CD4+ T cells to generate CAR-T cells, followed by viral transduction by FOXP3 [94]. It is important to note that the activity of CAR-Tregs has been shown to depend on the costimulatory domain [95]. Specifically, the 4-1BB domain of CAR-Tregs decreases their immunosuppressive function, while the CD28 domain maintains it and, in particular, stimulates the release of the anti-inflammatory cytokine IL-10 [95]. In addition, the CD28 costimulation domain is more effective in reducing the secretion of IL-2, GM-CSF, TNF-α, and IFN-γ from Teff cells and in suppressing the activity of these cells [95]. In the presence of inflammatory cytokines, CAR-Tregs have been found to reduce their stability and may alter their functional characteristics [96]. In particular, they can turn into Th17 cells that produce IL-17, so CAR-Tregs may be effective in treating SSc if the influence of inflammatory cytokines, such as IL6, IL21, and IFNɑ, which inhibit Treg activity, is eliminated [96].

Among the anti-CD19 CAR-T cells, the cell product KYV-101, derived from a fully human, anti-CD19, second-generation CAR construct Hu19-CD828Z, has been associated with decreased cytokine production, which is correlated with a lower risk of cytokine release syndrome (CRS) and reduced neurotoxicity in patients with B-cell lymphoma, according to the data from clinical trial NCT02659943 [97]. A special study confirmed that this cell construct exhibits CAR-mediated, CD19-dependent activity against autologous B-cells and comparably low inflammatory cytokine production when derived from cells from SLE, SSc, and idiopathic inflammatory myositis (IIM) patients [98]. This finding suggests that this cell construct may have potential for broad applications in the treatment of autoimmune diseases. This cell construct is also evaluated in a phase I/II open-label multicenter study (NCT06400303).

In addition to CRS and neurotoxicity, other side effects that may occur when anti-CD19 CAR-T cells are used to treat SSc must be considered. These side effects have been previously described when treating hematological malignancies [99,100]. CAR-T therapy is usually preceded by lymphodepleting chemotherapy to reduce endogenous lymphocytes and prepare a niche for the engraftment of CAR-T infusions, thereby supporting their long-term activity [101]. Fludarabine and cyclophosphamide are known to be the most efficient and most common lymphodepleting agents [101]. Data obtained from the treatment of relapsed and refractory B-cell malignancies indicate that the lymphodepleting chemotherapy regimen can cause early and late cytopenia, which manifests as neutropenia, thrombocytopenia, and anemia, and is a serious problem [102]. Several methods for mitigating the adverse effects of lymphodepletion procedures are outlined. For instance, the toxicity of lymphodepleting agents can be minimized by adhering to the optimal exposure calculated using special population modeling [103]. Using bendamustine for lymphodepletion in patients with refractory or relapsed large B-cell lymphoma has been found to reduce the incidence of cytopenia, compared with fludarabine/cyclophosphamide [104]. A comprehensive assessment of the safety of anti-CD19 CAR-T cell therapy should also consider that it may cause prolonged B-cell aplasia due to the depletion of both diseased and healthy B cells [99]. Aplasia-associated hypogammaglobulinemia may in turn elevate the risk of infection [99]. From this perspective, continuous patient monitoring is essential in order to identify the consequences of CAR-T cell therapy promptly. If necessary, immunoglobulin replacement therapy is prescribed, especially in cases of long-standing B-cell aplasia [105]. However, this is a standard procedure for pediatric patients with B-cell malignancies [106].

There are currently other clinical trials underway to assess the use of CAR-T cell therapy in the treatment of SSc (Table 1). A Phase I/II study is evaluating the safety and efficacy of CABA-201, a CD19 CAR-T cell therapy, in patients with active disease (NCT06328777). Also, a Phase I trial evaluating the safety and efficacy of anti-CD19 CAR-T cells to treat childhood-onset SSc (NCT06792344). Phase I clinical trial initiated to evaluate the safety and efficacy of CD19-BCMA CAR-T for the treatment of patients with refractory scleroderma (NCT05085444). The Phase II trial is designed to evaluate the safety and efficacy of CD19-BCMA CAR-T therapy in patients with multiple refractory autoimmune diseases, including SSc (NCT06794008). A Phase I clinical trial has begun to assess the efficacy, safety and cellular metabolism of CT1190B CAR-T cell therapy for treating patients with refractory or progressive SSc or moderate-to-severe refractory SLE (NCT06822881). A Phase I clinical trial has recently been initiated to study the preliminary efficacy and safety of universal allogeneic CD19/BCMA CAR-T cell therapy in patients with relapsing/refractory autoimmune diseases, including SSc (NCT06941129).

## 8. Conclusions and Future Perspectives

SSc is an autoimmune disease characterized by impaired immune system tolerance, leading to the development of autoreactive T and B lymphocyte clones. Despite efforts to find therapeutic approaches, the disease is still considered incurable. The clinical heterogeneity of the disease poses an additional challenge to treatment. Immunosuppressants have traditionally been used to treat SSc. These drugs can control the cutaneous and visceral fibrotic processes and remain the primary treatment option despite their serious side effects. Recently, a number of biological drugs with targeted action have been developed. These drugs can alleviate clinical symptoms; however, their effectiveness is limited, and they cannot improve patients’ conditions in the long term. The lack of effective SSc treatment methods underscores the need for new approaches that can restore immune tolerance and promote patient recovery. Data accumulated by researchers allows us to hope that cell-based therapy methods can provide such effective treatment for SS.

The possibility of using autologous HSCT to treat SSc has been investigated. Several clinical trials have demonstrated its effectiveness in controlling the disease long-term compared to standard immunosuppressants. However, severe complications and a high mortality rate after transplantation remain unresolved issues. Further research, refinement of the method, and careful patient selection are needed for its widespread implementation in clinical practice.

Of the various cell-based therapies studied, MSC therapy shows promise for treating SSc. MSCs have demonstrated potential as a regenerative therapy by restoring damaged tissues without causing apparent toxicity. Clinical trials have confirmed the safety and efficacy of MSC therapy in improving the clinical symptoms of SSc and stabilizing patients’ conditions, both locally and systemically. Local MSC therapy has been used to treat digital ulcers and fibrosis, improving both vascularization and elasticity. Systemic MSC therapy can treat diffuse skin thickening and fibrosis of internal organs, such as interstitial lung disease, which is one of the leading causes of death in patients with SSc. MSC therapy can complement standard drug treatment with immunosuppressants for refractory disease. Further research is necessary to enhance the sustainability of MSCs and understand the long-term effects of these cells in patients with SSc. Additionally, reducing the cost of the procedure is desirable for wider application of the method in the treatment of patients.

Another new potential approach is the use of T-cell therapy. Theoretically, polyclonal Tregs could be used to treat SSc because their function is suppressed in patients with the disease. Therefore, transplanting them could provide immune modulation and disease control. This method has been successfully used to treat other SAIDs, such as type 1 diabetes and SLE. However, problems with in vitro expansion and long-term persistence, as well as difficulties in identifying specific antigens, have slowed the introduction of Tregs into clinical practice. Significant advances in studying this approach for treating SSc have yet to be made. Also, significant progress has not yet been achieved in the use of TolDCs therapy in the treatment of SSc.

The engineering of T cells by creating CARs that recognize antigens independently of HLA has led to significant progress in treating hematological malignancies and has shown initial success in treating patients with autoimmune diseases. Targeting CAR-T cells to B cells that bear specific antigens, such as CD19, or dual targeting of CD19 on B cells and BCMA on long-lived plasma cells, can lead to the widespread depletion of autoantibody-producing cells in affected tissues. This restores normal function and resets the pathophysiological processes in SSc. CAR-T therapy studies using anti-CD19 CAR-T cells have demonstrated both safety and effectiveness in the treatment of SSc, with results achieved using both autologous and allogeneic CAR-T cells. These findings suggest that, under proper therapeutic conditions, this approach may hold promise for the treatment of SSc in clinical practice. CAR-T cell therapy has a significant advantage over other cell-based therapies discussed here due to the availability of the anti-CD19 CAR-T cell product, KYV-101. This product has demonstrated its effectiveness in destroying B-cells while producing a reduced level of cytokines. This minimizes side effects when used, such as the risk of developing CRS. The product will be ready for clinical practice in treating SSc once it has passed appropriate clinical trials. CAAR-T cell therapy is a promising type of CAR-T therapy, but the most relevant autoantigens to target must first be identified. Additionally, antigen-specific CAR-Tregs have several advantages over polyclonal Tregs: they are safer, can restore immunological tolerance, require fewer cells, and reduce the risk of nonspecific immune suppression. In the near future, it is possible that cell products for clinical use will emerge using these two types of CAR-T cells. CAR-T cell therapy is considered preferable to HSCT because it is equally effective but has fewer side effects. However, it should be noted that, at present, comparisons between CAR-T cell therapy and HSCT must be considered provisional. This is because the CAR-T data for SSc consists of isolated cases and early-phase trials. In contrast, large Phase II/III randomized trials and registry studies are available for HSCT. In terms of monitoring the safety of CAR-T cell therapy, it is important to consider the risk of developing B-cell aplasia and the subsequent hypogammaglobulinemia. However, reducing the toxicity of CAR-T cell therapy’s preparatory phase, lymphodepletion, is also important. Additionally, reducing the cost of the procedure is necessary so that it is accessible to a wide range of people around the world. As knowledge accumulates, a better understanding of the pathophysiological mechanisms of SSc will help develop optimal treatment strategies. While the search for effective new treatments is ongoing and more data are needed to determine which patients may benefit from which approach, it is clear that CAR-T cell therapy is one of the most promising areas of cell-based therapy for treating SSc.

## Data Availability

The original contributions presented in this study are included in the article/Supplementary Material. Further inquiries can be directed to the corresponding author.

## Supplementary Materials

The following supporting information can be downloaded at: https://www.mdpi.com/article/10.3390/cimb48010076/s1.
