# Supplementary material for: Emerging Cell-Based Therapies for Systemic Sclerosis: From Stem Cells to CAR-T Cells
Source: Curr Issues Mol Biol. 2026 Jan 12;48(1):76. doi: 10.3390/cimb48010076 (PMC12839790; doi:10.3390/cimb48010076)
Supplement: Supplementary file 1 [file cimb-48-00076-s001.zip › cimb-4010048-supplementary.pdf]

Supplementary

Table S1. Detailed summary of clinical evidence for cell-based therapies in systemic sclerosis (SSc). Outcomes and adverse events are reported as described in the corresponding peer-reviewed publication when available; otherwise, information is taken from the trial registry record (ClinicalTrials.gov; accessed 10 December 2025).

Therapy (product) – Trial ID	Study Type (Phase)	Patients (n)	Target/Mechanism	Clinical Outcome	Adverse Events (AEs)	Reference
Autologous HSCT–NCT00278525	Phase II	19 (diffuse SSc)	Autologous non-myeloablative HSCT vs cyclophosphamide (CYC).	10/10 HSCT patients improved (skin score vs 0/9 on CYC. 8 of 9 CYC patients progressed (↑mRSS or ↓FVC), whereas none progressed after HSCT. Improvements in skin and pulmonary function with HSCT persisted through 2 years.	No treatment-related mortality (TRM). HSCT conditioning side effects (mucositis, neutropenia) occurred but were manageable. Overall, HSCT was well-tolerated; fewer serious infections than anticipated, given non-myeloablative regimen.	19
Autologous HSCT vs CYC (“SCOT”) – NCT00114530	PhaseII/III	75 (diffuse SSc)	Autologous CD34⁺ HSCT (myeloablative, CD34-selected) vs 12 × monthly CYC.	HSCT significantly improved event-free survival and overall survival – survival ~94% HSCT vs 81% CYC at 4.5 yrs. HSCT patients had lasting improvements in skin and lung fibrosis, whereas CYC patients deteriorated. Benefits sustained at 6–11 years follow-up.	TRM ~6% with HSCT (1 early death within 2 yrs). HSCT arm had higher acute toxicity (transient cytopenias, infections), but long-term safety acceptable; no graft-vs-host(autologous). Cyclophosphamide arm had no treatment-related deaths but more relapses. Notably, HSCT’s survival advantage outweighed its upfront risks.	17
Autologous HSCT (EBMT “NISSC-2” registry) – NCT02516124	Prospective non-interventional stydy	82	Autologous HSCT (various conditioning).	High 2-year progression-free survival (81.8%) and overall survival (90%) post-HSCT. Clinical responses in ~89% (skin scores improved in 55 evaluable patients; mean mRSS ↓; 88.7% achieved response). Lung function stabilized or	100-day TRM ~5% (4/80 patients) – lower than historic studies. Improved patient selection and CD34⁺ graft selection contributed to safety. Beyond early transplant risk, no unexpected	21

				improved (FVC ↑ in 37 pts).	late effects observed.	
Autologous HSCT	Retrospective clinical study (single-center)	17 SSc patients (severe, refractory)	Broad immune reset; high-dose cyclophosphamide + ATG conditioning to eliminate autoreactive immune cells.	Median 9.1-year follow-up: Skin fibrosis improved (mRSS↓ from 31 to 7), lung function stabilized/improved in 87% of patients; 63% off immunosuppressants post-HSCT. Lung function stabilized or improved in 12 of 15 evaluable patients; 86% had improved gastrointestinal symptoms. All surviving patients reported substantially improved quality of life, and 63% remained off immunosuppressive therapy post-HSCT. 5-year survival ~94% in severe SSc.	Treatment-related mortality (TRM) ~5.8% (1 early post-transplant death)	24
Umbilical cord-derived allogeneic MSCs (“CARE-SSc” trial) – NCT04356287	Phase I/II randomized trial	18 (diffuse cutaneous SSc)	Will compare single vs repeated MSC infusions vs placebo.	Trial in progress (double-blind). No outcomes available yet; study completion expected April 2025	Not reported yet. A 2023 interim safety review showed no infusion-related serious AEs. MSC therapy expected to have a favorable safety profile.	ClinicalTrials.gov (NCT04356287; accessed 10 December 2025)
Allogeneic MSCs NCT00962923	Phase I/II open-label study	14 (diffuse SSc)	Exploration of safety and efficacy of allogeneic MSCs.	Skin fibrosis markedly improved: mean mRSS↓ from 20.1 to 13.8 at 12 months. 69% of patients achieved skin score reduction. Lung function remained stable (no FVC decline). Benefits sustained at mean 15.6 months follow-up.	No serious AEs attributable to MSC therapy; no immunogenic reactions reported; no infections or malignancies observed over follow-up. One patient had transient fever after infusion.	43
Autologous MSCs - adipose-derived regenerative cells NCT02396238	Randomized double-blind Phase III trial (STAR)	88 SSc patients (48 ADRC vs 40 placebo)	Locally delivered ADRCs (from fat) to repair tissue and modulate immunity in hands (improve vascular regeneration	At 48 weeks, hand-function scores improved. Diffuse SSc subset showed a trend toward greater improvement. Overall, >50% of ADRC-treated diffuse SSc patients achieved clinically important hand	ADRC therapy was well tolerated. Small-volume fat harvest and cell injection were feasible and safe; no significant treatment-related AEs.	44

			and fibrosis in fingers).	function improvement vs 16% of placebo.		
Allogeneic bone marrow MSCs	Phase I/II open-label trial	19 SSc patients (severe diffuse)	Single MSC infusion. Post-infusion immune profiling was performed. 14/19 patients were defined as responders (improved skin/organ status).	Responders showed a rise in IL-10-producing regulatory B cells and increased B-cell IL-10 gene expression shortly after MSC infusion. Non-responders had pre-treatment B cells with higher pro-fibrotic cytokines (IL-6, TGF-β). In vitro, MSC directly increased IL-10 production by B cells.	No infusion reactions; therapy was well tolerated. No significant AEs reported in this study beyond transient mild injection-site or infusion-related events.	30
IFN-γ + TNF-α preconditioned MSCs	Preclinical (mouse model of SSc)	N/A (BLM-induced SSc model in mice)	Pretreated MSCs with pro-inflammatory cytokines to enhance immunosuppressive function. Targeted pathogenic macrophages via suppression of CCL2 chemokine.	MSCs inhibited recruitment of profibrotic monocytes/macrophages by reducing CCL2 production. This led significantly reduced skin thickening and collagen deposition, lowered α-SMA and TGF-β1 levels, and increased apoptosis resistance in skin.	MSC-treated mice tolerated therapy without obvious AEs.	34
Allogeneic MSCs	Retrospective matched-cohort study	333 (113 -MSCT group; 220 – control)	Immunomodulatory and pro-regenerative effects of MSCs on survival.	Improved survival in MSC group (≈89% vs 73% in controls). After propensity-score matching, MSCT still conferred a survival benefit (10-year survival ~88% vs 80%). MSCT showed ~60% reduction in mortality risk on multivariate analysis. Subgroup analysis suggested greatest benefit in younger patients and those with severe SSc.	No TRM for MSCT. MSC therapy was generally well-tolerated, no major AEs were noted. MSC infusions have a favorable safety profile, with minimal immediate infusion reactions and no significant long-term AEs reported in this cohort.	45
Autologous regulatory T-cells (Treg) therapy – NCT05214014	Phase II randomized trial (Treg vs std. care)	25 (15 Treg, 10 control	Polyclonal $CD4^+CD25^+$ Tregs to study immune suppression/tolerance.	At 6 months post-infusion, skin elasticity improved significantly in the Treg-treated group with no further skin thickening. Quality-of-life also improved vs baseline. Treg therapy halted	No severe treatment-related AEs reported. Treg infusions were well-tolerated; no infections or malignancies observed during 6-month follow-up.	65

				disease progression in all treated patients, whereas controls showed ongoing fibrosis progression.		
Regulatory T Cells (Treg expansion via low-dose IL-2 (LD-IL2)	Phase I/IIa open-label trial (TRANS REG Basket)	9	LD-IL2 at 1×10^6 IU/day to selectively expand Tregs, restoring immune balance.	After LD-IL2 therapy, Treg frequency increased ~1.8-fold by day 8. Other lymphocyte subsets (Teff, B cells) unchanged. At 6 months, clinical disease markers (mRSS , HAQ, lung function) remained stable, indicating no progression.	The therapy was well tolerated. No serious AEs and significant toxicity. Minor injection-site reactions and transient flu-like symptoms were reported in some patients, but overall safety was excellent.	68
Regulatory T Cells (Treg expansion via LD-IL2)	Controlled clinical study (open-label, parallel groups)	46 SSc patients (23 IL-2 + std. care vs 23 std. care)	LD-IL2 at 5.0 $\times 10^5$ IU/day for 5 days added to standard immunosuppressive therapy, aiming to expand Tregs and correct Th17/Treg imbalance.	At 24 weeks, LD-IL2-treated patients showed a significant rise in Treg counts, restoring the Th17/Treg ratio toward normal. lD-IL2 patients achieved more reduction in mRSS and symptom severity than controls.	LD-IL2 was well tolerated and safety with no obvious AEs reported. No patients developed infections or cytokine reactions attributable to LD-IL2.	67
KYV-101 – autologous CD19 CAR-T NCT06400303	Phase I/II open-label trial (ongoing)	21 (planned)	CD19 on B cells (CAR T–mediated B-cell depletion)	Ongoing (no results yet). Expected deep B-cell depletion and immune reset in refractory SSc.	Not yet reported (trial recruiting). Potential CAR-T toxicities (CRS, neurotoxicity) anticipated, but none observed so far in SSc	ClinicalTrials.gov (NCT06400303; accessed 10 December 2025)
Allogeneic CD19/BCMA CAR-T NCT06941129	Phase I investigator-initiated trial (new)	12 (multiple autoimmune diseases)	CD19 & BCMA (B cells and plasma cells	Newly launched trial for refractory SSc and other autoimmune diseases. Not yet recruiting as of 2025.	No data yet (study start 2025). Allogeneic "off-the-shelf" CAR-T expected to carry risk of graft-versus-host and typical CAR-T toxicities; safety to be evaluated.	ClinicalTrials.gov (NCT06941129; accessed 10 December 2025)
Dual CD19/BCMA CAR-T NCT05085444	early phase I trial (ongoing)	9 (estimated)	CD19 & BCMA (dual-target CAR-T)	Enrollment ongoing (init. 2021). No efficacy readouts yet.	Safety is primary focus. Trial in progress – no data disclosed to date.	ClinicalTrials.gov (NCT05085444; accessed 10 December 2025)
CT1190B CAR-T (for SLE/SSc) – NCT06822881	Phase I dose-escalation trial (planned)	27 (SLE and SSc)	CD19-targeted CAR-T (allogeneic "off-the-shelf" T cells)	Trial will evaluate clinical response in refractory SLE and SSc. No outcomes yet.	No data yet. Trial will monitor infusion reactions, CRS, etc.	ClinicalTrials.gov (NCT06822881; accessed 10 December 2025)

Anti-CD19 CAR-T (juvenile SSc) – NCT06792344	Phase I pilot (pediatric SSc, ongoing)	12	Anti-CD19 CAR-T cell depletion of B cells	Ongoing study in childhood-onset diffuse SSc. No published results yet.	No data has been reported.	ClinicalTrials.gov (NCT06792344; accessed 10 December 2025)
Dual CD19/BCMA CAR-T – NCT06794008	Phase 2 trial (planned)	50 (multiple diseases, including SSc)	CD19 & BCMA (dual CAR targets for B cells/plasma cells)	No data available yet.	No information yet. Prior phase I suggested manageable safety; phase 2 will further assess toxicity profile.	ClinicalTrials.gov (NCT06794008; accessed 10 December 2025)
Universal allogeneic CD19 CAR-T NCT05859997	Phase I pilot (case series published)	3 treated (2 SSc, 1 myositis)	Anti-CD19 CAR-T cells generated by CRISPR/Cas9	B-cell depletion was observed in 2 weeks after treatment. Two diffuse SSc patients achieved marked improvement in clinical measurements, including decreased autoantibodies. Significant reduction of inflammation and fibrosis observed on imaging.	Treatment was well-tolerated. No CRS or neurotoxicity noted after 6 months follow-up.	78
CABA-201 – autologous CD19 CAR T – NCT06328777 (RESET-SSc)	Phase 1/2 open-label trial (recruiting)	12 (planned: ≥6 skin, 6 organ)	A single dose of CABA-201 in combination with CYC and fludarabine will be evaluated.	No data has been reported.	No information about AEs.	ClinicalTrials.gov (NCT06328777; accessed 10 December 2025)
Autologous CD19 CAR-T Cells targeting B cells	Case report (first-in-disease use)	1 patient with diffuse, refractory SSc	CD19 CAR-T to deplete B cells and reset autoimmunity.	Skin fibrosis, Carpal arthritis was improved, pulmonary fibrosis remained stable on follow-up of 3 months. Tender joint counts improved from n=22 at baseline to n=3 after CAR-T infusion. ANA and RP11 autoantibodies were not detectable on follow-up of 3 and 6 months.	The CAR-T therapy was well tolerated; the patient experienced no CRS or other serious adverse reaction. This single-case result suggests CD19 CAR-T was safe and effective in refractory SSc.	69
CD19 CAR-T Cells	Case series (pilot study)	1 (Scl70+ and progressive pulmonary fibrosis)	pre-existing therapy with mycophenolate or nintedanib before CD19 CAR-T cell infusion (3rd-generation).	After therapy the fingers were less puffy. Skin fibrosis reduced (mRSS↓). Pulmonary fibrosis and lung function were also improved. No digital ulcers occurred. CRP, hsTNT, ANA and	No AEs were detected.	76

				Scl70 titres normalised or strongly decreased.		
CAR-NK (iPSC-derived) – Off-the-shelf NK cells (QN-139b)	Case report (first-in-human)	1 (severe, diffuse cutaneous SSc)	Dual CAR targeting CD19 & BCMA (elimination of B cells and plasma cells)	Marked clinical improvement at 6 months: reversal of fibrosis with restored skin elasticity and vascular structure; significant reduction in autoantibody levels and a shift towards naive B-cell phenotype. Inflammation was suppressed and tissue repair/regeneration observed.	Minimal toxicity; no CRS or GVHD noted. No genomic abnormalities detected in infused CAR-NK cells.	79

Notes: "Patients (n)" indicates treated participants when reported in the publication; otherwise it reflects planned enrollment from the registry. Abbreviations: mRSS, modified Rodnan skin score; FVC, forced vital capacity; DLCO, diffusing capacity for carbon monoxide; CRS, cytokine release syndrome; ICANS, immune effector cell–associated neurotoxicity syndrome; TRM, treatment-related mortality; GVHD, graft-versus-host disease.
